# Beneficial effects of green tea: A literature review

**DOI:** 10.1186/1749-8546-5-13

**Published:** 2010-04-06

**Authors:** Sabu M Chacko, Priya T Thambi, Ramadasan Kuttan, Ikuo Nishigaki

**Affiliations:** 1 NPO International Laboratory of Biochemistry, 1-166 Uchide, Nakagawa-ku, Nagoya, 454-0926, Japan; 2 Amala Cancer Research Center, Amala Nagar, Thrissur, Kerala, 680 555, India

## Abstract

The health benefits of green tea for a wide variety of ailments, including different types of cancer, heart disease, and liver disease, were reported. Many of these beneficial effects of green tea are related to its catechin, particularly (-)-epigallocatechin-3-gallate, content. There is evidence from *in vitro *and animal studies on the underlying mechanisms of green tea catechins and their biological actions. There are also human studies on using green tea catechins to treat metabolic syndrome, such as obesity, type II diabetes, and cardiovascular risk factors.

Long-term consumption of tea catechins could be beneficial against high-fat diet-induced obesity and type II diabetes and could reduce the risk of coronary disease. Further research that conforms to international standards should be performed to monitor the pharmacological and clinical effects of green tea and to elucidate its mechanisms of action.

## Background

In recent years, the health benefits [1] of consuming green tea, including the prevention of cancer [2] and cardiovascular diseases [3], the anti-inflammatory [4], antiarthritic [5], antibacterial [6], antiangiogenic [7], antioxidative [8], antiviral [9], neuroprotective [10], and cholesterol-lowering effects [11] of green tea and isolated green tea constituents are under investigation. However, adding green tea to the diet may cause other serious health concerns.

The health-promoting effects of green tea are mainly attributed to its polyphenol content [12], particularly flavanols and flavonols, which represent 30% of fresh leaf dry weight [1]. Recently, many of the aforementioned beneficial effects of green tea were attributed to its most abundant catechin, (-)-epigallocatechin-3-gallate (EGCG) [13-15]. Green tea extracts are more stable than pure epigallocatechin gallate, one of the major constituents of green tea, because of the presence of other antioxidant constituents in the extract [8]. In general, herbal medicines are complex mixtures of different compounds that often act in a synergistic fashion to exert their full beneficial effect [11]. However, relatively few herbal medicines have been well characterized and their efficacy demonstrated in systematic clinical trials as compared to Western drugs. This review article highlights the recent research on the efficacy, action mechanisms, and side effects of green tea and its catechins in *in vitro*, *in vivo*, and *ex vivo *systems [16].

The review on green tea and its catechins focused on language literature in English. The literature search was conducted in the following databases: Pubmed (1980-2009), EMBASE (1980-2009), Allied and complementary Medicine Database (AMED, 1985-2009) and China Journals Full Text Database (1975-2009). The keywords used were selected from the following terms: green tea, catechins, anticancer, diabetes, polyphenols, *in vivo *studies, general pharmacology and toxicology. The health benefits and adverse effects of green tea and its catechins were reviewed.

The authors read full articles and reached consensus after discussion. Articles included in the study covered the following effects of green tea: (1) the health benefits in humans and animals, (2) absorption of metal ions and drug-metabolizing enzymes, (3) antioxidation and inhibition of oxidative stress, (4) carbohydrate metabolism and diabetes mellitus, and (5) adverse effects. A total of 105 peer-reviewed papers in English were selected for this review.

### Green tea

Tea is one of the most popular beverages consumed worldwide. Tea, from the plant *Camellia sinensis*, is consumed in different parts of the world as green, black, or Oolong tea. Among all of these, however, the most significant effects on human health have been observed with the consumption of green tea [17]. The first green tea was exported from India to Japan during the 17th century. It is estimated that about 2.5 million tons of tea leaves are produced each year throughout the world, with 20% produced as green tea, which is mainly consumed in Asia, some parts of North Africa, the United States, and Europe [18]. The association between tea consumption, especially green tea, and human health has long been appreciated [19,20]. Green tea and black tea are processed differently during manufacturing. To produce green tea, freshly harvested leaves are immediately steamed to prevent fermentation, yielding a dry, stable product. This steaming process destroys the enzymes responsible for breaking down the color pigments in the leaves and allows the tea to maintain its green color during the subsequent rolling and drying processes. These processes preserve natural polyphenols with respect to the health-promoting properties. As green tea is fermented to Oolong and then to black tea, polyphenol compounds (catechins) in green tea are dimerized to form a variety of theaflavins, such that these teas may have different biological activities.

### Green tea composition

The chemical composition of green tea is complex: proteins (15-20% dry weight), whose enzymes constitute an important fraction; amino acids (1-4% dry weight) such as theanine or 5-*N-*ethylglutamine, glutamic acid, tryptophan, glycine, serine, aspartic acid, tyrosine, valine, leucine, threonine, arginine, and lysine; carbohydrates (5-7% dry weight) such as cellulose, pectins, glucose, fructose, and sucrose; minerals and trace elements (5% dry weight) such as calcium, magnesium, chromium, manganese, iron, copper, zinc, molybdenum, selenium, sodium, phosphorus, cobalt, strontium, nickel, potassium, fluorine, and aluminum; and trace amounts of lipids (linoleic and α-linolenic acids), sterols (stigmasterol), vitamins (B, C, E), xanthic bases (caffeine, theophylline), pigments (chlorophyll, carotenoids), and volatile compounds (aldehydes, alcohols, esters, lactones, hydrocarbons). Due to the great importance of the mineral presence in tea, many studies have determined their levels in tea leaves and their infusions (Table 1) [21]. Fresh leaves contain, on average, 3-4% of alkaloids known as methylxanthines, such as caffeine, theobromine, and theophylline [22]. In addition, there are phenolic acids such as gallic acids and characteristic amino acid such as theanine present [22].

**Table 1:** Composition (%) of green tea, black tea, and black tea infusion [21]

Compound	Green Tea*	Black tea*	Infusion*
Protein	15	15	trace
Amino acids	4	4	3.5
Fiber	26	26	0
Others carbohydrates	7	7	4
Lipids	7	7	trace
Pigments	2	2	trace
Minerals	5	5	4.5
Phenolic compounds^‡^	30	5	4.5
Oxidized phenolic compounds^§^	0	25	4.5

* Data refer to dry weight of tea leaves.

^† ^Infusion time: 3 minutes

^‡ ^Especially flavonoids

^§ ^Especially thearubigins and theaflavins

Green tea contains polyphenols, which include flavanols, flavandiols, flavonoids, and phenolic acids; these compounds may account for up to 30% of the dry weight. Most of the green tea polyphenols (GTPs) are flavonols, commonly known as catechins. Products derived from green tea are mainly extracts of green tea in liquid or powder form that vary in the proportion of polyphenols (45-90%) and caffeine content (0.4-10%). The major flavonoids of green tea are various catechins, which are found in greater amounts in green tea than in black or Oolong tea [23]. There are four kinds of catechins mainly find in green tea: epicatechin, epigallocatechin, epicatechin-3-gallate, and EGCG [24]. The preparation methods influence the catechins both quantitatively and qualitatively; the amount of catechins also varies in the original tea leaves due to differences in variety, origin, and growing conditions [25]. The preparation of fresh green tea cannot totally extract catechins from the leaves; therefore, the concentration found differs from the absolute values determined through the complete extraction of leaves [26]. Moreover, catechins are relatively unstable and could be quantitatively and qualitatively modified during the time frame of an experiment [27,28]. Thus, comparison of ingested doses in animal studies is not possible because the catechin quantification before administration is often not known.

### Health benefits of green tea in humans and animals

Studies using animal models show that green tea catechins provide some protection against degenerative diseases [29]. Some studies indicated that green tea has an antiproliferative activity on hepatoma cells and a hypolipidemic activity in hepatoma-treated rats, as well as the prevention of hepatoxicity [29] and as a preventive agent against mammary cancer post-initiation [29]. Green tea catechins could also act as antitumorigenic agents [30] and as immune modulators in immunodysfunction caused by transplanted tumors or by carcinogen treatment [29]. Moreover, green tea, its extract, and its isolated constituents were also found to be effective in preventing oxidative stress [31] and neurological problems [32].

Green tea consumption has also been linked to the prevention of many types of cancer, including lung, colon, esophagus, mouth, stomach, small intestine, kidney, pancreas, and mammary glands [33]. Several epidemiological studies and clinical trials showed that green tea (and black and Oolong teas to a lesser extent) may reduce the risk of many chronic diseases [34]. This beneficial effect has been attributed to the presence of high amounts of polyphenols, which are potent antioxidants. In particular, green tea may lower blood pressure and thus reduce the risk of stroke and coronary heart disease. Some animal's studies suggested that green tea might protect against the development of coronary heart disease by reducing blood glucose levels and body weight [35]. However, all these data are based on middle-aged animals' populations, not the elderly populations, which nutritional status tends to be more adversely influenced by age-related biological and socioeconomic factors [36].

Tea components possess antioxidant, antimutagenic, and anticarcinogenic effects and could protect humans against the risk of cancer by environmental agents [37]. Sano *et al*. [38] reported the inhibitory effects of green tea leaves against tert-butyl hydroperoxide-induced lipid peroxidation, and a similar antioxidant effect on the kidney was observed after oral administration of the major tea polyphenol EGCG. The antioxidative potency of crude catechin powder and individual catechins was tested in experiments using the active oxygen method. Crude catechins reduced the formation of peroxides far more effectively than dl-α-tocopherol [39]. Shim *et al*. [40] studied the chemopreventive effect of green tea among cigarette smokers and found that it can block the cigarette-induced increase in sister chromatid exchange frequency.

The effectiveness of green tea in treating any type of diarrhea and typhoid has been known in Asia since ancient times [41-43]. Green tea catechins have an inhibitory effect on *Helicobacter pylori *infection [44,45]. Effects of green tea against the influenza virus, especially in its earliest stage, as well as against the *Herpes simplex *virus have also been demonstrated [46-48]. Furthermore, Weber *et al*. [9] observed that adenovirus infection is inhibited *in vitro *by green tea catechins.

In humans, Hirasawa and Takada [49] studied the antifungal activity of green tea catechins against *Candida albicans *and the convenience of a combined treatment with catechins and lower doses of antimycotics, which may help to avoid the side effects of antimycotics. Green tea consumption has also been associated with increased bone mineral density, and it has been identified as an independent factor protecting against the risk of hip fractures; this effect was considered independent of smoking status, hormone replacement therapy, coffee drinking, and the addition of milk to tea [50]. Park *et al*. [51] observed the positive effects of green tea extracts and GTPs on the proliferation and activity of bone cells. The proliferation of hepatic stellate cells is closely related to the progression of liver fibrosis in chronic liver diseases, and EGCG has a potential inhibitory effect on the proliferation of these cells [52,53]. Green tea strengthens the immune system action because it protects it against oxidants and radicals. Recent studies suggested that GTPs might protect against Parkinson's and Alzheimer's diseases and other neurodegenerative diseases [10,54]. Studies have demonstrated GTP neuroprotectant activity in cell cultures and animal models, such as the prevention of neurotoxin-induced cell injury [54]. Green tea is considered to be useful for insect stings due mainly to its anti-inflammatory effects and its capacity to stop bleeding [55,56]. Some studies have suggested an inverse association between green tea consumption and the risk of kidney stone formation [41,57]. In an experimental cataractogenesis system, green tea acted by preserving the antioxidant defense system of the lens [58]. Skrzydlewska *et al*. [59] indicated a beneficial effect of green tea in alcohol intoxication. In addition to all of these reported properties, which have helped the recognition of green tea as functional food by some authors [60], green tea is also currently used in the preparation of a variety of foods, pharmaceutical preparations, dentifrices, and cosmetics [61].

Tea has been shown anticarcinogenic effects against breast cancer in experimental studies [62]. However, epidemiologic evidence that tea protects against breast cancer has been inconsistent [62]. A case-control study was conducted in southeastern China between 2004 and 2005 [63]. The incidence cases were 1009 female patients aged 20-87 years with histologically confirmed breast cancer, and the 1009 age-matched controls were healthy women randomly recruited from breast disease clinics. Information on duration, frequency, quantity, preparation, and type of tea consumption as well as diet and lifestyle were collected by face-to-face interviews using a validated and reliable questionnaire. In comparison with non-tea drinkers, green tea drinkers tended to reside in urban settings, to have more education, and to consume more coffee, alcohol, soy, vegetables, and fruits. After adjusting established and potential confounding factors, green tea consumption was associated with a reduced risk of breast cancer. Similar dose-response relationships were observed for duration of drinking green tea, number of cups consumed, and new batches prepared per day.

Hsu *et al*. [64] demonstrated the effects of supplementation with decaffeinated green tea extract (catechins) on hemodialysis-induced reactive oxygen species, atherosclerotic disease risk factors, and proinflammatory cytokines. The pharmacokinetics of one oral dose of catechins was compared between healthy subjects and hemodialysis patients. The authors compared the antioxidant effects of three different doses (0, 455, and 910 mg) of oral catechins with that of oral vitamin C (500 mg) during a hemodialysis session. In patients, catechin supplementation reduced hemodialysis-enhanced plasma hypochlorous acid activity more effectively than did placebo or vitamin C. Between the treatments with 455 and 910 mg catechins, no significant difference was found in the reduction of plasma hypochlorous acid activity. Catechins also significantly reduced proinflammatory cytokine expression enhanced by hemodialysis.

### Effects on absorption of metal ions

Tea catechins can affect iron absorption, particularly in groups at risk of iron deficiency [65,66], but their effects on other ions are poorly understood. Green tea ingestion over a long period does not affect the apparent absorption of copper, whereas it decreases that of zinc and increases that of manganese [67]. However, catechin intake does not affect the plasma concentration of these ions [68]. Green tea catechins have the potential to affect absorption and metabolism of ions because flavonoids interact with a variety of metal ions [69].

### Effects on drug-metabolizing enzymes

Long-term ingestion of green tea increases UDP-glucuronosyl transferase activity in rats [66,70,71], and after being absorbed, catechins are metabolized by drug-metabolizing enzymes in various organs [72,73]. Thus, the increased glucuronidation through UDP-glucuronosyl transferase induction is postulated to contribute to the anticarcinogenic effect of green tea by facilitating the metabolism of chemical carcinogens into inactive products that are readily excreted. The interaction between 2-amino-3-methylimidazol (4,5-f)quinoline (IQ) and green tea catechin metabolism was examined [74]. IQ is a precarcinogen that was originally detected in an extract of fried meat. The major route of IQ biotransformation in rats is cytochrome P450 in the first step, followed by conjugation to a sulfate and a glucuronide conjugate. Green tea modifies IQ metabolism in rats, increasing the formation of IQ glucuronides, which are then excreted in the urine. Moreover, protection against cancers induced by polycyclic aromatic hydrocarbons by green tea catechins may be due to the inhibition of their cytochrome P450 metabolism, but the effect of green tea on cytochrome P450 enzymes depends on the particular form. The long-term consumption of green tea increases cytochrome P450 1A1 and 1A2 activities, but not 2B1 and 2E1 activities, in normal rats. However, it is difficult to draw conclusions about a beneficial effect of green tea against carcinogens involving only modulation of this metabolic pathway.

### Effects on antioxidant markers and oxidative stress

Green tea is a popular neutraceutical as an antioxidant. Antioxidants are compounds that protect cells against the damaging effects of reactive oxygen species, such as singlet oxygen, superoxide, peroxyl radicals, hydroxyl radicals, and peroxynitrite. An imbalance between antioxidants and reactive oxygen species results in oxidative stress, leading to cellular damage [75]. Catechins are hypothesized to help protect against these diseases by contributing, along with antioxidant vitamins (i.e., vitamins C and E) and enzymes (i.e., superoxide dismutase and catalase), to the total antioxidant defense system [76].

*In vivo *studies showed that green tea catechins increase total plasma antioxidant activity [77,78]. Intake of green tea extracts also increases the activity of superoxide dismutase in serum and the expression of catalase in the aorta; these enzymes are implicated in cellular protection against reactive oxygen species [78,79]. This action is combined with direct action on oxygen species by a decrease in the nitric oxide plasma concentration [80]. Malondialdehyde, a marker of oxidative stress, also decreases after green tea intake [77,80]. These results suggest that catechins could have a direct (antioxidant) or indirect (increase of activity or expression) effect. Since catechins can act as antioxidants *in vitro*, they might prevent the oxidation of other antioxidants, such as vitamin E. However, ingestion of green tea catechins does not modify the plasma status of vitamins E and C *in vivo *[78,81,82]. Nevertheless, one study reported that catechins increase vitamin E concentration in low-density lipoprotein [81] and in this way could protect low-density lipoprotein against peroxidation [77].

Pilipenko *et al*. [83] assessed the tolerance of tableted green tea and its effect on the antioxidant status indices. Twenty-five patients with different gastrointestinal pathologies were included in the study and divided into treatment and control groups. The tolerance of tableted green tea was good in the treatment group, who showed better dynamics of quality-of-life indices, especially in scales of body pain and social functioning. There were no significant differences in biochemical analysis between the groups, which may indicate the safety of this product. Analysis revealed that the treatment group showed a decreased level of all antioxidant status indices, as reflected in a significant decreasing of the lipid peroxidation index from 4.63 to 4.14.

### Effects on carbohydrate metabolism

Type II diabetes is a heterogeneous disorder that involves resistance of glucose and lipid metabolism in peripheral tissues to the biological activity of insulin and inadequate insulin secretion by pancreatic β cells [84]. Animal models of diabetes are available: Zucker rats, which are genetically obese; injection of streptozotocin or alloxan, which destroys pancreatic β cells; and treatment with sucrose-rich diets, which induces obesity and insulin resistance.

In a study by Sabu *et al*. [85], administration of GTPs (500 mg/kg) to normal rats increased glucose tolerance significantly at 60 minutes. GTPs were also found to reduce significantly serum glucose levels in alloxan diabetic rats at a dose of 100 mg/kg. Continued daily administration (15 days) of the extract at 50 or 100 mg/kg produced 29% and 44% reduction, respectively, in the elevated serum glucose level produced by alloxan administration. Elevated hepatic and renal enzymes produced by alloxan were found to be reduced significantly by GTPs. The serum lipid peroxidation level was increased by alloxan and reduced significantly by the administration of 100 mg/kg of GTPs. Decreased liver glycogen resulting from alloxan administration showed a significant increase after GTP treatment. The GTP-treated group showed increased antioxidant potential, as seen from improvements in superoxide dismutase and glutathione levels. However, catalase, lipid peroxidation, and glutathione peroxidase levels were unchanged. These results indicate that alterations in the glucose utilizing system and oxidation status in rats that were increased by alloxan were partially reversed by the administration of GTPs [85].

Catechins also reduced plasma triglyceride levels in an oral glucose-tolerance test in normal rats [86]. Green tea extract intake reduced these values in both Zucker rats and rats fed a sucrose-rich diet [87,88]. Several human- and animal-based studies suggested that green tea and its flavonoids have antidiabetic effects [86,89,90]. Green tea flavonoids were also shown to have insulin-like activities [91] as well as insulin-enhancing activity [92].

The antihyperglycemic effect of black tea was reported by Gomes *et al*. [93]. EGCG was found to inhibit intestinal glucose uptake by the sodium-dependent glucose transporter SGLT1, indicating its increase in controlling blood sugar [94]. Streptozotocin diabetic rats showed increased sensitivity to platelet aggregation and thrombosis, and this abnormality could be improved by dietary catechins from green tea [95,96]. Alloxan produces oxygen radicals in the body, which cause pancreatic injury [75] and are responsible for increased blood sugar.

Under *in vivo *conditions, glutathione acts as an antioxidant, and its decrease was reported in a diabetes mellitus model [97]. The increased glutathione content in the liver of the rats treated with GTPs may be one of the factors responsible for the inhibition of lipid peroxidation. Superoxide dismutase and catalase are the two major scavenging enzymes that remove the toxic free radicals *in vivo*. Vucic *et al*. [98] reported that the activity of superoxide dismutase is low in diabetes mellitus.

The Mediterranean Islands (MEDIS) epidemiological study is a cross-sectional health and nutrition survey that aims to evaluate the association between various sociodemographic, bioclinical, dietary, and other lifestyle habits and the prevalence of the common cardiovascular disease risk factors (i.e., hypertension, dyslipidemia, diabetes, and obesity) among elderly people without a history of any chronic disease and living in the Mediterranean islands. Because data relating tea consumption with clinical characteristics are lacking in elderly populations, in the context of the MEDIS study, the authors sought to evaluate whether green tea consumption is independently associated with fasting blood glucose levels and the prevalence of type II diabetes mellitus [99]. An earlier study was aimed at providing evidence of improvement in glucose metabolism in diabetic mice and healthy humans upon green tea consumption [35]. Green tea promoted glucose metabolism in healthy human volunteers at 1.5 g/kg as shown in oral glucose-tolerance tests. Green tea also lowered blood glucose levels in diabetic db+/db+ mice and streptozotocin-diabetic mice two to six hours after administration at 300 mg/kg without affecting serum insulin level, whereas no effect was observed in control mice (+m/+m and normal ddY mice).

### Effect of EGCG on diabetes

A study by Waltner-Law *et al*. [91] provided compelling *in vitro *evidence that EGCG decreases glucose production of H4IIE rat hepatoma cells. The investigators showed that EGCG mimics insulin, increases tyrosine phosphorylation of the insulin receptor and the insulin receptor substrate, and reduces gene expression of the gluconeogenic enzyme phosphoenolpyruvate carboxykinase. Recently, green tea and green tea extracts were demonstrated to modify glucose metabolism beneficially in experimental models of type II diabetes mellitus [35,100]. In addition, EGCG ameliorates cytokine-induced β cell damage *in vitro *[101] and prevents the decrease of islet mass induced by treatment with multiple low doses of streptozotocin *in vivo *[102].

Lambert *et al*. [103] showed that intragastric administration of EGCG at a dose of 75 mg/kg resulted in a Cmax of 128 mg/l total plasma EGCG and a terminal half-life of 83 minutes. Furthermore, in humans an oral intake of EGCG at a dose of 50 mg (0.7 mg/kg) resulted in a Cmax of 130 mg/l total plasma EGCG and a terminal half-life of 112 minutes [104]. These results indicate that rodents must be orally administered 100- to 600-fold more EGCG (depending on whether they are administered by gavage or by feed admixture) to achieve similar plasma concentrations as those found in humans. Total plasma EGCG concentrations shown to be efficacious in mice and rats can be reached by an intake of low to moderate doses of EGCG in humans.

### Effect on obesity

The effects of tea on obesity and diabetes have received increasing attention. Tea catechins, especially EGCG, appear to have antiobesity and antidiabetic effects [105]. African black tea extract has been shown to suppress the elevation of blood glucose during food intake and reduce the body weight in KK-A(y)/TaJcl diabetic mice [106]. Although few epidemiological and clinical studies have shown the health benefits of EGCG on obesity and diabetes, the mechanisms of its actions are emerging based on various laboratory data. These mechanisms may be related to certain pathways, such as through the modulations of energy balance, endocrine systems, food intake, lipid and carbohydrate metabolism, and redox status [88].

A double-blind, placebo-controlled, cross-over design study showed that consumption of a beverage containing green tea catechins, caffeine, and calcium increases 24-h energy expenditure by 4.6%, but the contribution of the individual ingredients could not be distinguished. It was suggested that such modifications were sufficient to prevent weight gain. It has been reported that the body weights of rats and their plasma triglyceride, cholesterol, and low-density lipoprotein cholesterol were significantly reduced by feedings of Oolong, black, and green tea leaves to the animals. In addition, the inhibition of growth and suppression of lipogenesis in MCF-7 breast cancer cells may be through down-regulation of fatty acid synthase gene expression in the nucleus and stimulation of cell energy expenditure in the mitochondria [107,108]. When fed to mice, EGCG purified from green tea decreased diet-induced obesity in mice by decreasing energy absorption and increasing fat oxidation [109]. The increased and prolonged sympathetic stimulation of thermogenesis by the interaction between polyphenols and caffeine could be of value in assisting the management of obesity [110].

Recent data from human studies indicate that the consumption of green tea and green tea extracts may help reduce body weight, mainly body fat, by increasing postprandial thermogenesis and fat oxidation. In a randomized, double-blind, placebo-controlled, cross-over pilot study, six overweight men were given 300 mg EGCG per day for two days. Fasting and postprandial changes in energy expenditure and substrate oxidation were assessed. Resting energy expenditure did not differ significantly between EGCG and placebo treatments, although during the first postprandial monitoring phase, respiratory quotient values were significantly lower with EGCG treatment compared to the placebo. These findings suggest that EGCG alone has the potential to increase fat oxidation in men and may thereby contribute to the antiobesity effects of green tea. However, more studies with a greater sample size and a broader range of age and body mass index are needed to define the optimal dose [111].

### Adverse effects of green tea

Although green tea has several beneficial effects on health, the effects of green tea and its constituents may be beneficial up to a certain dose yet higher doses may cause some unknown adverse effects. Moreover, the effects of green tea catechins may not be similar in all individuals. EGCG of green tea extract is cytotoxic, and higher consumption of green tea can exert acute cytotoxicity in liver cells, a major metabolic organ in the body [112]. Another study found that higher intake of green tea might cause oxidative DNA damage of hamster pancreas and liver [113]. Yun *et al*. [114] clarified that EGCG acts as a pro-oxidant, rather than an antioxidant, in pancreatic β cells *in vivo*. Therefore, high intake of green tea may be detrimental for diabetic animals to control hyperglycemia. At a high dose (5% of diet for 13 wk), green tea extract induced a thyroid enlargement (goiter) in normal rats [115,116]. This high-level treatment modified the plasma concentrations of the thyroid hormones. However, drinking even a very high dietary amount of green tea would be unlikely to cause these adverse effects in humans.

Harmful effects of tea overconsumption (black or green) are due to three main factors: (1) its caffeine content, (2) the presence of aluminum, and (3) the effects of tea polyphenols on iron bioavailability. Green tea should not be taken by patients suffering from heart conditions or major cardiovascular problems. Pregnant and breast-feeding women should drink no more than one or two cups per day, because caffeine can cause an increase in heart rhythm. It is also important to control the concomitant consumption of green tea and some drugs, due to caffeine's diuretic effects [117]. Some studies revealed the capacity of tea plants to accumulate high levels of aluminum. This aspect is important for patients with renal failure because aluminum can be accumulated by the body, resulting in neurological diseases; it is therefore necessary to control the intake of food with high amounts of this metal [118]. Likewise, green tea catechins may have an affinity for iron, and green tea infusions can cause a significant decrease of the iron bioavailability from the diet [119].

## Conclusions

Laboratory studies showed the health effects of green tea. As the human clinical evidence is still limited, future research needs to define the actual magnitude of health benefits, establishes the safe range of tea consumption associated with these benefits, and elucidates the mechanisms of action. Development of more specific and sensitive methods with more representative models along with the development of good predictive biomarkers will give a better understanding of how green tea interacts with endogenous systems and other exogenous factors. Definitive conclusions concerning the protective effect of green tea have to come from well-designed observational epidemiological studies and intervention trials. The development of biomarkers for green tea consumption, as well as molecular markers for its biological effects, will facilitate future research in this area.

## Abbreviations

EGCG: epigallocatechin-3-gallate; GTPs: green tea polyphenols; UDP: Uridine di-phospatase; IQ: 2-amino-3-methylimidazol (4,5-f)quinoline; MEDIS: Mediterranean Islands; SDLT: Sodium dependent glucose transporter; AMED: Allied and complementary Medicine Database.

## Competing interests

The authors declare that they have no competing interests.

## Authors' contributions

SMC and PTT did the literature search and drafted the manuscript. RK and IN critically reviewed the literature and revised the manuscript. All authors read and approved the final version of the manuscript.

## References

[B1] McKay DL Blumberg JB The role of tea in human health: An update J Am Coll Nutr 2002 21 1 13 11838881 10.1080/07315724.2002.10719187

[B2] Kavanagh KT Hafer LJ Kim DW Mann KK Sherr DH Rogers AE Sonenshein GE Green tea extracts decrease carcinogen-induced mammary tumor burden in rats and rate of breast cancer cell proliferation in culture J Cell Biochem 2001 82 387 398 10.1002/jcb.1164 11500915

[B3] Sueoka N Suganuma M Sueoka E Okabe S Matsuyama S Imai K Nakachi K Fujiki H A new function of green tea: prevention of lifestyle-related diseases Ann N Y Acad Sci 2001 928 274 280 11795518 10.1111/j.1749-6632.2001.tb05656.x

[B4] Dona M Dell'Aica I Calabrese F Benelli R Morini M Albini A Garbisa S Neutrophil restraint by green tea: inhibition of inflammation, associated angiogenesis, and pulmonary fibrosis J Immunol 2003 170 4335 4341 12682270 10.4049/jimmunol.170.8.4335

[B5] Haqqi TM Anthony DD Gupta S Ahmad N Lee MS Kumar GK Mukhtar H Prevention of collagen-induced arthritis in mice by a polyphenolic fraction from green tea Proc Natl Acad Sci USA 1999 96 4524 4529 10.1073/pnas.96.8.4524 10200295 PMC16365

[B6] Sudano Roccaro A Blanco AR Giuliano F Rusciano D Enea V Epigallocatechin-gallate enhances the activity of tetracycline in staphylococci by inhibiting its efflux from bacterial cells Antimicrob Agents Chemother 2004 48 1968 1973 10.1128/AAC.48.6.1968-1973.2004 15155186 PMC415601

[B7] Sartippour MR Shao ZM Heber D Beatty P Zhang L Liu C Ellis L Liu W Go VL Brooks MN Green tea inhibits vascular endothelial growth factor (VEGF) induction in human breast cancer cells J Nutr 2002 132 2307 2311 12163680 10.1093/jn/132.8.2307

[B8] Osada K Takahashi M Hoshina S Nakamura M Nakamura S Sugano M Tea catechins inhibit cholesterol oxidation accompanying oxidation of low density lipoprotein *in vitro* Comp Biochem Physiol Part C Toxicol Pharmacol 2001 128 153 164 10.1016/S1532-0456(00)00192-7 11239828

[B9] Weber JM Ruzindana-Umunyana A Imbeault L Sircar S Inhibition of adenovirus infection and adenain by green tea catechins Antiviral Res 2003 58 167 173 10.1016/S0166-3542(02)00212-7 12742577

[B10] Weinreb O Mandel S Amit T Youdim MBH Neurological mechanisms of green tea polyphenols in Alzheimer's and Parkinson's diseases J Nutr Biochem 2004 15 506 516 10.1016/j.jnutbio.2004.05.002 15350981

[B11] Raederstorff DG Schlachter MF Elste V Weber P Effect of EGCG on lipid absorption and plasma lipid levels in rats J Nutr Biochem 2003 14 326 332 10.1016/S0955-2863(03)00054-8 12873714

[B12] Naghma K Hasan M Tea polyphenols for health promotion Life Sciences 2007 81 519 533 10.1016/j.lfs.2007.06.011 17655876 PMC3220617

[B13] Moyers SB Kumar NB Green tea polyphenols and cancer chemoprevention: multiple mechanisms and endpoints for phase II trials Nutr Rev 2004 62 204 211 10.1111/j.1753-4887.2004.tb00041.x 15212320

[B14] Mandel S Weinreb O Amit T Youdim MB Cell signaling pathways in the neuroprotective actions of the green tea polyphenol(-)-epigallocatechin-3-gallate: implications for neurodegenerative diseases J Neurochem 2004 88 1555 1569 15009657 10.1046/j.1471-4159.2003.02291.x

[B15] Higdon JV Frei B Tea catechins and polyphenols: health effects, metabolism, and antioxidant functions Crit Rev Food Sci Nutr 2003 43 89 143 10.1080/10408690390826464 12587987

[B16] Xiang YZ Shang HC Gao XM Zhang BL A comparison of the ancient use of ginseng in traditional Chinese medicine with modern pharmacological experiments and clinical trials Phytother Res 2008 22 7 851 858 10.1002/ptr.2384 18567057

[B17] Cabrera C Artacho R Giménez R Beneficial effects of green tea: a review J Am Coll Nutr 2006 25 79 99 16582024 10.1080/07315724.2006.10719518

[B18] Japanese Green Tea Online.com http://www.japanesegreenteaonline.com

[B19] Weisburger JH Approaches for chronic disease prevention based on current understanding of underlying mechanisms Am J Clin Nutr 2000 71 6 1710S 1714S 10837325 10.1093/ajcn/71.6.1710S

[B20] Sato T Miyata G The nutraceutical benefit, part I: green tea Nutrition 2000 16 315 317 10.1016/S0899-9007(99)00301-9 10758375

[B21] Belitz DH Grosch W Quı'mica de los Alimentos 1997 Zaragoza: Acribia

[B22] Graham HN Green tea composition, consumption, and polyphenol chemistry Prev Med 1992 21 334 350 10.1016/0091-7435(92)90041-F 1614995

[B23] Vinson JA Black and green tea and heart disease: a review Biofactors 2000 13 127 132 10.1002/biof.5520130121 11237171

[B24] Sano M Tabata M Suzuki M Degawa M Miyase T Maeda-Yamamoto M Simultaneous determination of twelve tea catechins by high-performance liquid chromatography with electrochemical detection Analyst 2001 126 816 820 10.1039/b102541b 11445943

[B25] Khokhar S Magnusdottir SGM Total phenol, catechin, and caffeine contents of teas commonly consumed in the United Kingdom J Agric Food Chem 2002 50 565 570 10.1021/jf010153l 11804530

[B26] Fernandez PL Martin MJ Gonzalez AG Pablos F HPLC determination of catechins and caffeine in tea. Differentiation of green, black and instant teas Analyst 2000 125 421 425 10.1039/a909219f 10829341

[B27] Chen ZY Zhu QY Wong YF Zhang Z Chung HY Stabilizing effect of ascorbic acid on green tea catechins J Agr Food Chem 1998 46 2512 2516 10.1021/jf971022g

[B28] Chen ZY Zhu QY Tsang D Huang Y Degradation of green tea catechins in tea drinks J Agr Food Chem 2001 49 477 482 10.1021/jf000877h 11170614

[B29] Vanessa C Gary W A Review of the Health Effects of Green Tea Catechins in In Vivo Animal Models J Nutr 2004 134 3431S 3440S 15570050 10.1093/jn/134.12.3431S

[B30] Roomi MW Ivanov V Kalinovsky T Niedzwiecki A RathIn M In vitro and in vivo antitumorigenic activity of a mixture of lysine, proline, ascorbic acid, and green tea extract on human breast cancer lines MDA-MB-231 and MCF-7 Medical Oncol 2007 22 2 129 138 10.1385/MO:22:2:129 15965275

[B31] Babu PV Sabitha KE Shyamaladevi CS Therapeutic effect of green tea extract on oxidative stress in aorta and heart of streptozotocin diabetic rats Chem Biol Interact 2006 162 114 120 10.1016/j.cbi.2006.04.009 16860299

[B32] Unno K Takabayashi F Yoshida H Choba D Fukutomi R Kikunaga N Kishido T Oku N Hoshino M Daily consumption of green tea catechin delays memory regression in aged mice Biogerontology 2007 8 2 89 95 10.1007/s10522-006-9036-8 16957869

[B33] Koo MWL Cho CH Pharmacological effects of green tea on the gastrointestinal system Eur J Pharmacol 2004 500 177 185 10.1016/j.ejphar.2004.07.023 15464031

[B34] Zaveri NT Green tea and its polyphenolic catechins: medicinal uses in cancer and noncancer applications Life Sci 2006 78 2073 2080 10.1016/j.lfs.2005.12.006 16445946

[B35] Tsuneki H Ishizuka M Terasawa M Wu JB Sasaoka T Kimura I Effect of green tea on blood glucose levels and serum proteomic patterns in diabetic (db/db) mice and on glucose metabolism in healthy humans BMC Pharmacol 2004 4 18 21 10.1186/1471-2210-4-18 15331020 PMC517497

[B36] Meydani M Nutrition interventions in aging and age associated disease Ann N Y Acad Sci 2001 928 226 235 11795514 10.1111/j.1749-6632.2001.tb05652.x

[B37] Mukhtar H Wang ZY Katlya SK Agarwal R Tea components: antimutagenic and anticarcinogenic effects Prev Med 1992 21 351 360 10.1016/0091-7435(92)90042-G 1614996

[B38] Sano M Takahashi Y Yoshino K Shimoi K Nakamura Y Tomita I Oguni I Konomoto H Effect of tea (*Camellia sinensis *L.) on lipid peroxidation in rat liver and kidney: a comparison of green and black tea feeding Biol Pharm Bull 1995 18 1006 1008 7581239 10.1248/bpb.18.1006

[B39] Hara Y Advances in Food Science and Technology Nippon Shokuhin Kogyo 1990 Tokyo: Gakkai: Korin

[B40] Shim JS Kang MH Kim YH Roh JK Roberts C Lee IP Chemopreventive effect of green tea (*Camellia sinensis*) among cigarette smokers Cancer Epidemiol Biomarkers 1995 4 387 391 7655335

[B41] McKay DL Blumberg JB The role of tea in human health: an update J Am Coll Nutr 2002 21 1 13 11838881 10.1080/07315724.2002.10719187

[B42] Lu H Meng X Li C Sang S Patten C Sheng S Hong J Bai N Winnik B Ho CT Yang CS Glucuronides of tea catechins: enzymology of biosynthesis and biological activities Drug Metab Dispos 2003 31 452 461 10.1124/dmd.31.4.452 12642472

[B43] Wu CH Lu FH Chang CS Chang TC Wang RH Chang CJ Relationship among habitual tea consumption, percent body fat, and body fat distribution Obes Res 2003 11 1088 1095 10.1038/oby.2003.149 12972679

[B44] Takabayashi F Harada N Yamada M Murohisa B Oguni I Inhibitory effect of green tea catechins in combination with sucralfate on *Helicobacter pylori *infection in Mongolian gerbils J Gastroenterol 2004 39 61 63 10.1007/s00535-003-1246-0 14767736

[B45] Yee YK Koo MWL Szeto ML Chinese tea consumption and lower risk of *Helicobacter *infection J Gastroenterol Hepatol 2002 17 552 555 10.1046/j.1440-1746.2002.02718.x 12084028

[B46] Toda M Okubo S Ohnishi R Shimamura T Antibacterial and bactericidal activities of Japanese green tea Nippon Saikingaku Zasshi 1989 44 669 672 2677434 10.3412/jsb.44.669

[B47] Mukoyama A Ushijima H Nishimura S Koike H Toda M Hara Y Shimamura T Inhibition of rotavirus and enterovirus infections by tea extracts Jpn J Med Sci Biol 1991 44 181 186 1668240 10.7883/yoken1952.44.181

[B48] Yama TS Shaha S Hamilton-Millera JMT Microbiological activity of whole and fractionated crude extracts of tea (*Camellia sinensis*), and of tea components FEMS Microbiol Lett 1997 152 169 174 10.1111/j.1574-6968.1997.tb10424.x 9228784

[B49] Hirasawa M Takada K Multiple effects of green tea catechin on the antifungal activity of antimycotics against *Candida albicans* J Antimicrob Chemother 2004 53 225 229 10.1093/jac/dkh046 14688042

[B50] Muraki S Yamamoto S Ishibashi H Horiuchi T Hosoi T Suzuki T Orimo H Nakamura K Green tea drinking is associated with increased bone mineral density J Bone Miner Res 2003 18 S241

[B51] Park H Ko S Kim J Kim S Effects of green tea extracts and polyphenols on the proliferation and activity of bone cells J Bone Miner Res 2003 18 S342

[B52] Dorchies OM Wagner S Waldhauser KM Buetler TM Ruegg UT Anti-fibrotic properties of green tea catechins on mouse muscle cell cultures Neuromuscul Disord 2003 13 639

[B53] Sakata R Ueno T Nakamura T Sakamoto M Torimura T Sata M Green tea polyphenols epigallocatechin-3-gallate inhibits platelet-derived growth factor-induced proliferation of human hepatic stellate cell line LI90 J Hepatol 2004 40 52 59 10.1016/S0168-8278(03)00477-X 14672614

[B54] Pan TH Jankovic J Le WD Potential therapeutic properties of green tea polyphenols in Parkinson's disease Drugs Aging 2003 20 711 721 10.2165/00002512-200320100-00001 12875608

[B55] Sagesaka-Mitane Y Miwa M Okada S Platelet aggregation inhibitors in middle aged Japanese men and women Ann Epidemiol 1998 7 280 284

[B56] Dvorakova K Dorr RT Valcic S Timmermann B Alberts DS Pharmacokinetics of the green tea derivative, EGCG, by the topical route of administration in mouse and human skin Cancer Chemother Pharmacol 1999 43 331 335 10.1007/s002800050903 10071985

[B57] Ishizuk H Eguchi H Oda T Ogawa S Nakagawa K Honjo S Kono S Relation of coffee, green tea, and caffeine intake to gallstone disease in middle-age Japanese men Eur J Epidemiol 2003 18 401 405 10.1023/A:1024237927985 12889685

[B58] Gupta SK Halder N Srivastava S Trivedi D Joshi S Varma SD Green tea (*Camellia sinensis*) protects against selenite-induced oxidative stress in experimental cataractogenesis Ophthalmic Res 2002 34 258 263 10.1159/000063881 12297700

[B59] Skrzydlewska E Ostrowska J Stankiewicz A Farbiszewski R Green tea as a potent antioxidant in alcohol intoxication Addict Biol 2002 7 307 314 10.1080/13556210220139523 12126490

[B60] Ferrari CKB Torres EAFS Biochemical pharmacology of functional foods and prevention of chronic diseases of aging Biomed Pharmacother 2003 57 251 260 10.1016/S0753-3322(03)00032-5 12888262

[B61] Arburjai T Natsheh FM Plants used in cosmetics Phytother Res 2003 17 987 1000 10.1002/ptr.1363 14595575

[B62] Min Zhang C D'Arcy JH Jiang-ping H Xing X Green tea and the prevention of breast cancer: a case-control study in Southeast China Carcinogenesis 2005 28 5 1074 1078 10.1093/carcin/bgl252 17183063

[B63] Zhang M Holman CDAJ Huang JP Xie X Green tea and the prevention of breast cancer: a case-control study in southeast China Carcinogenesis 2008 29 8 1594 1600 10.1093/carcin/bgn129 17183063

[B64] Hsu SP Wu MS Yang CC Huang KC Liou SY Hsu SM Chien CT Chronic green tea extract supplementation reduces hemodialysis-enhanced production of hydrogen peroxide and hypochlorous acid, atherosclerotic factors, and proinflammatory cytokines Am J Clin Nutr 2007 86 5 1539 1547 17991670 10.1093/ajcn/86.5.1539

[B65] Samman S Sandstrom B Toft MB Bukhave K Jensen M Sorensen SS Hansen M Green tea or rosemary extract added to foods reduces nonheme-iron absorption Am J Clin Nutr 2001 73 607 612 11237939 10.1093/ajcn/73.3.607

[B66] Nelson M Poulter J Impact of tea drinking on iron status in the UK: a review J Hum Nutr Diet 2004 17 43 54 10.1046/j.1365-277X.2003.00497.x 14718031

[B67] Deng Z Tao B Li X He J Chen Y Effect of green tea and black tea on the metabolisms of mineral elements in old rats Biol Trace Elem Res 1998 65 75 86 10.1007/BF02784115 9877538

[B68] Record IR McInerney JK Dreosti IE Black tea, green tea, and tea polyphenols: effects on trace element status in weanling rats Biol Trace Elem Res 1996 53 27 43 10.1007/BF02784542 8862735

[B69] Mira L Fernandez MT Santos M Rocha R Florencio MH Jennings KR Interactions of flavonoids with iron and copper ions: a mechanism for their antioxidant activity Free Radic Res 2002 36 1199 1208 10.1080/1071576021000016463 12592672

[B70] Maliakal PP Coville PF Wanwimolruk S Tea consumption modulates hepatic drug metabolizing enzymes in Wistar rats J Pharm Pharmacol 2001 53 569 577 10.1211/0022357011775695 11341376

[B71] Sohn OS Surace A Fiala ES Richie JP Jr Colosimo S Zang E Weisburger JH Effects of green and black tea on hepatic xenobiotic metabolizing systems in the male F344 rat Xenobiotica 1994 24 119 127 10.3109/00498259409043226 8017087

[B72] Donovan JL Crespy V Manach C Morand C Besson C Scalbert A Remesy C Catechin is metabolized by both the small intestine and liver of rats J Nutr 2001 131 1753 1757 11385063 10.1093/jn/131.6.1753

[B73] Okushio K Suzuki M Matsumoto N Nanjo F Hara Y Methylation of tea catechins by rat liver homogenates Biosci Biotechnol Biochem 1999 63 430 432 10.1271/bbb.63.430 10192923

[B74] Embola CW Weisburger JH Weisburger MC Urinary excretion of N-OH-2-amino-3-methylimidazo [4,5-f]quinoline-N-glucuronide in F344 rats is enhanced by green tea Carcinogenesis 2001 22 1095 1098 10.1093/carcin/22.7.1095 11408354

[B75] Halliwell B Gutteridge JMC Free Radicals in Biology and Medicine 1985 Oxford: Clarendon Press 10.1016/0748-5514(85)90028-5 3939136

[B76] Abdel-Raheim MAM Enas AH Khaled AE Effect of green tea extract and vitamin c on oxidant or antioxidant Indian J Clin Biochem 2009 24 3 280 287 10.1007/s12291-009-0053-7 PMC3453301 23105850

[B77] Yokozawa T Nakagawa T Kitani K Antioxidative activity of green tea polyphenol in cholesterol-fed rats J Agric Food Chem 2002 50 3549 3552 10.1021/jf020029h 12033827

[B78] Skrzydlewska E Ostrowska J Farbiszewski R Michalak K Protective effect of green tea against lipid peroxidation in the rat liver, blood serum and the brain Phytomedicine 2002 9 232 238 10.1078/0944-7113-00119 12046864

[B79] Negishi H Xu JW Ikeda K Njelekela M Nara Y Yamori Y Black and green tea polyphenols attenuate blood pressure increases in stroke-prone spontaneously hypertensive rats J Nutr 2004 134 38 42 14704290 10.1093/jn/134.1.38

[B80] Yokozawa T Nakagawa T Lee KI Cho EJ Terasawa K Takeuchi S Effects of green tea tannin on cisplatin-induced nephropathy in LLC-PK1 cells and rats J Pharm Pharmacol 1999 51 1325 1331 10.1211/0022357991776912 10632092

[B81] Tijburg LBM Wiseman SA Meijer GW Weststrate JA Effects of green tea, black tea and dietary lipophilic antioxidants on LDL oxidizability and atherosclerosis in hypercholesterolaemic rabbits Atherosclerosis 1997 135 37 47 10.1016/S0021-9150(97)00139-1 9395271

[B82] Alessio HM Hagerman AE Romanello M Carando S Threlkeld MS Rogers J Dimitrova Y Muhammed S Wiley RL Consumption of green tea protects rats from exercise-induced oxidative stress in kidney and liver Nutr Res 2003 22 1177 1188 10.1016/S0271-5317(02)00421-9

[B83] Pilipenko VI Shakhovskaia AK Mal'tsev GIU Isakov VA Influence of tableted green tea on index the antioxidant status patients with disease digestion organs Vopr Pitan 2008 77 4 58 62 18839809

[B84] Del Prato S Piero M Riccardo CB Phasic Insulin Release and Metabolic Regulation in Type 2 Diabetes Diabetes 2007 51 S109 10.2337/diabetes.51.2007.S109 11815468

[B85] Sabu MC Smitha K Kuttan R Anti-diabetic activity of green tea polyphenols and their role in reducing oxidative stress in experimental diabetes J Ethnopharmacol 2002 83 109 116 10.1016/S0378-8741(02)00217-9 12413715

[B86] Wu LY Juan CC Ho LT Hsu YP Hwang LS Effect of green tea supplementation on insulin sensitivity in Sprague-Dawley rats J Agric Food Chem 2004 52 643 648 10.1021/jf030365d 14759162

[B87] Hasegawa N Yamda N Mori M Powdered green tea has antilipogenic effect on Zucker rats fed a high-fat diet Phytother Res 2003 17 477 480 10.1002/ptr.1177 12748982

[B88] Yang MH Wang CH Chen HL Green, Oolong and black tea extracts modulate lipid metabolism in hyperlipidemia rats fed high-sucrose diet J Nutr Biochem 2001 12 14 20 10.1016/S0955-2863(00)00140-6 11179857

[B89] Iso H Date C Wakai K Fukui M Tamakoshi A The relationship between green tea and total caffeine intake and risk for self-reported type 2 diabetes among Japanese adults Ann Intern Med 2006 144 554 562 16618952 10.7326/0003-4819-144-8-200604180-00005

[B90] Wolfram S Raederstorff D Preller M Wang Y Teixeira SR Riegger C Weber P Epigallocatechin gallate supplementation alleviates diabetes in rodents J Nutr 2006 136 3512 3518 10.1093/jn/136.10.2512 16988119

[B91] Waltner-Law ME Wang XL Law BK Hall RK Nawano M Granner DK Epigallocatecin gallate, a constituent of green tea, represses hepatic glucose production J Biol Chem 2002 277 34933 34940 10.1074/jbc.M204672200 12118006

[B92] Anderson RA Polansky MM Tea enhances insulin activity J Agric Food Chem 2002 50 7182 7186 10.1021/jf020514c 12428980

[B93] Gomes A Vedasiromoni JR Das M Sharma RM Ganguly DK Anti-hyperglycemic effect of black tea (*Camellia sinensis*) in rat J Ethnopharmacol 1995 45 223 226 10.1016/0378-8741(95)01223-Z 7623488

[B94] Kobayashi Y Suzuki M Satsu H Arai S Hara Y Suzuki K Miyamoto Y Shimizu M Green tea polyphenols inhibit the sodium-dependent glucose transporter of intestinal epithelial cells by a competitive mechanism J Agric Food Chem 2000 48 5618 5623 10.1021/jf0006832 11087528

[B95] Yang JA Choi JH Rhee SJ Effects of green tea catechin on phospholipase A2 activity and antithrombus in streptozotocin diabetes rats J Nutr Sci Vitaminol (Tokyo) 1999 45 337 346 10524352 10.3177/jnsv.45.337

[B96] Choi JH Cha BK Rhee SJ Effect of green tea catechin on hepatic microsomal phospholipase A2 activities and changes of hepatic phospholipid species in streptozotocin-induced diabetic rats J Nutr Sci Vitaminol (Tokyo) 1998 44 673 683 9919487 10.3177/jnsv.44.673

[B97] Illing EKB Gray CH Lawrence RD Blood glutathione and non-glucose reducing substances in diabetes J Biochem 1951 48 637 640 10.1042/bj0480637 PMC1275389 14838916

[B98] Vucic M Gavell M Bozikov V Ashcroft JH Rocic B Superoxide dismutase activity in lymphocytes and polymorphonuclear cells of diabetic patients Eur J Clin Chem Biochem 1997 35 517 521 10.1515/cclm.1997.35.7.517 9263727

[B99] Polychronopoulos E Panagiotakos DB Polystipioti A Diet, lifestyle factors and hypercholesterolemia in elderly men and women from Cyprus Lipids Health Dis 2005 4 17 21 10.1186/1476-511X-4-17 16144549 PMC1208941

[B100] Wu LY Juan CC Hwang LS Hsu YP Ho PH Ho LT Green tea supplementation ameliorates insulin resistance and increases glucose transporter IV content in a fructose-fed rat model Eur J Nutr 2004 43 116 124 10.1007/s00394-004-0450-x 15083319

[B101] Han MK Epigallocatechin gallate, a constituent of green tea, suppresses cytokine-induced pancreatic beta-cell damage Exp Mol Med 2003 35 136 139 12754418 10.1038/emm.2003.19

[B102] Song EK Hur H Han MK Epigallocatechin gallate prevents autoimmune diabetes induced by multiple low doses of streptozotocin in mice Arch Pharm Res 2003 26 559 563 10.1007/BF02976881 12934649

[B103] Lambert JD Lee MJ Lu H Meng X Hong JJJ Seril DN Sturgill MG Yang CS Epigallocatechin-3-gallate is absorbed but extensively glucuronidated following oral administration to mice J Nutr 2003 133 4172 4177 14652367 10.1093/jn/133.12.4172

[B104] Ullmann U Haller J Decourt JP Girault N Girault J Richard-Caudron AS Pineau B Weber P A single ascending dose study of epigallocatechin gallate in healthy volunteers J Int Med Res 2003 31 88 101 12760312 10.1177/147323000303100205

[B105] Kao YH Chang HH Lee MJ Chen CL Tea, obesity, and diabetes Mol Nutr Food Res 2006 50 2 188 210 10.1002/mnfr.200500109 16416476

[B106] Shoji Y Nakashima H Glucose-lowering effect of powder formulation of African black tea extract in KK-A(y)/TaJcl diabetic mouse Arch Pharmacol Res 2006 29 9 786 794 10.1007/BF02974080 17024853

[B107] Rudelle S Ferruzzi MG Cristiani I Moulin J Mace K Acheson KJ Tappy L Effect of a thermogenic beverage on 24-hour energy metabolism in humans Obesity 2007 15 2 349 355 10.1038/oby.2007.529 17299107

[B108] Lin JK Lin-Shiau SY Mechanisms of hypolipidemic and anti-obesity effects of tea and tea polyphenols Mol Nutr Food Res 2006 50 2 211 217 10.1002/mnfr.200500138 16404708

[B109] Klaus S Pultz S Thone-Reineke C Wolfram S Epigallocatechin gallate attenuates diet-induced obesity in mice by decreasing energy absorption and increasing fat oxidation Int J Obes 2005 29 6 615 623 10.1038/sj.ijo.0802926 15738931

[B110] Dulloo AG Seydoux J Girardier L Chantre P Vandermander J Green tea and thermogenesis: interactions between catechin-polyphenols, caffeine and sympathetic activity Int J Obes Relat Metab Disord 2000 24 2 252 258 10.1038/sj.ijo.0801101 10702779

[B111] Boschmann M Thielecke F The effects of epigallocatechin-3-gallate on thermogenesis and fat oxidation in obese men: a pilot study J Am Coll Nutr 2007 26 4 389S 395S 17906192 10.1080/07315724.2007.10719627

[B112] Schmidt M Schmitz HJ Baumgart A Guedon D Netsch MI Kreuter MH Schmidlin CB Schrenk D Toxicity of green tea extracts and their constituents in rat hepatocytes in primary culture Food Chem Toxicol 2005 43 307 314 10.1016/j.fct.2004.11.001 15621343

[B113] Takabayashi F Tahara S Kanerko T Harada N Effect of green tea catechins on oxidative DNA damage of hamster pancreas and liver induced by N-nitrosobis (2-oxopropyl) amine and/or oxidized soybean oil Biofactors 2004 21 335 337 10.1002/biof.552210165 15630222

[B114] Yun SY Kim SP Song DK Effects of (*-*)-epigallocatechin-3-gallate on pancreatic beta-cell damage in streptozotocin-induced diabetic rats Eur J Pharmacol 2006 541 115 121 10.1016/j.ejphar.2006.04.040 16765345

[B115] Sakamoto Y Mikuriya H Tayama K Takahashi H Nagasawa A Yano N Yuzawa K Ogata A Aoki N Goitrogenic effects of green tea extract catechins by dietary administration in rats Arch Toxicol 2001 75 591 596 10.1007/s00204-001-0286-6 11808919

[B116] Satoh K Sakamoto Y Ogata A Nagai F Mikuriya H Numazawa M Yamada K Aoki N Inhibition of aromatase activity by green tea extract catechins and their endocrinological effects of oral administration in rats Food Chem Toxicol 2002 40 925 933 10.1016/S0278-6915(02)00066-2 12065214

[B117] Bruneton J Pharmacognosie. Phytochimie. Plantes Me'dicinales 2001 Paris: Technique Documentation-Lavoisier

[B118] Costa LM Gouveia ST Nobrega JA Comparison of heating extraction procedures for Al, Ca, Mg and Mn in tea samples Ann Sci 2002 18 313 318 10.2116/analsci.18.313 11918191

[B119] Hamdaoui MH Chabchob S Heidhili A Iron bioavailability and weight gains to iron-deficient rats fed a commonly consumed Tunisian meal "bean seeds ragout" with or without beef and with green or black tea decoction J Trace Elem Med Biol 2003 17 159 164 10.1016/S0946-672X(03)80020-2 14968927

